# European Correspondence

**Published:** 1876-10

**Authors:** 


### EUROPEAN CORRESPONDENCE.

*Editor Atlanta Medical and Surgical Journal:*

Dear Sir—My time has been so completely occupied, that I have not had an opportunity of writing you before; besides, before so doing, I wished to be able to give you some definite idea of the medical men and hospitals of this great city of London. Nothing of very great importance transpired during my voyage that would iterest you. Going direct from Rome,

Ga., to New York, and having an opportunity to sail, I only remained there two days. Your letters secured me an introduction to some of the most prominent medical men of that city, and among the number, Dr. J. Marion Sims, the kind hospitalities of whose home and table I had both the honor and pleasure of enjoying. Besides, he gave me advice and assistance in such a manner that I have been able to prosecute my studies much more favorably than I could otherwise have done.

Embarking in a vessel of the White Star Line, I bid adieu to New York and to my native land. Moving from the shore out on the bosom of the "billowy deep," with the spires of the receding city growing smaller and smaller, my mind was filled with feelings mingled with both pleasure and sadness. Save the occasional war-whoop of an Indian on board, the passing of a ship, the spouting of whales in the distance, the blowing of the whistle when enveloped in a fog, or the stern voice of the officer in charge, ringing out in the midnight stillness, nothing transpired to break the monotony of the voyage. I was on the ocean a few hours over eight days, and in two weeks from the time I left home, was settled down here and engaged at work. By making close connection in New York, the time from Atlanta to either London or Paris is about eleven days.

I soon found that board was as cheap as in Atlanta, and books can be purchased for a great deal less. Briefly, I will attempt to give you some idea of a portion of the hospitals, and their mode of teaching, there being too many to even attempt a description of them all. There are seven or eight leading schools and hospitals, called by the general name of "hospital." Each of these has a faculty composed of some of the best men in London. Each has a medical class and a systematic course of lectures on all the general and special branches. Some of the buildings and medical classes are larger than others, yet I do not think they materially differ. At each the didactic lectures continue between nine and ten months of the year, and the clinical teaching the entire year. While it is the custom of some of the leading physicians to leave London for two or three weeks during August, yet they alternate, a few going at a time, while the remaining officers and assistants fill their places, ennabling by this means the surgery and clinical work to continue the same. These general hospitals contain between seven hundred and one thousand beds each, divided into wards, each one being devoted to certain branches, viz: venereal, surgery, nervous troubles, diseases of women, heart, lungs, infectious diseases, etc. Each hospital has a most magnificent museum. A brief description of Guys will probably be a fair estimate of all. The models of human anatomy in wax in this, amounting to hundreds, must be seen, rather than described, to be appreciated. Besides the models in wax, the museum contains about two thousand preparations of human anatomy in the form of injected and preserved dissections. The museum of comparative anatomy contains over two thousand specimens, and that of pathological anatomy over five thousand, with more than two thousand drawings corresponding to those of the preparations; besides these there is an extensive branch in wax, containing several hundred specimens, representing various diseases of the skin. Each of these several branches has a guide-book giving a short description of the specimen and its pathological condition. It seems that one could almost learn medicine and surgery from these museums alone, as almost everything is represented. The number of out-patients daily seen at these hospitals is simply enormous. It is here that this densely populated city, with its "rolling seas" of living humanity, with its busied millions, offers to the medical student a variety of cases in any specialty he may desire. St. Bartholomew's alone treated last year 143,000 out-patients, which I suppose is about a fair average of all. You would probably be surprised to see the general cleanliness and dress of these patients. The large out-patient departments not only embrace general medical and surgical practice, but have special arrangements for the treatment of patients with special diseases. The medical teaching is different from ours, and the time required for study much longer. The students are required to attend the college and hospital practice for four years before they are allowed their degrees, and even then these degrees are very hard to obtain. The first two years they devote principally to the study of the primary branches, viz: anatomy, physiology, chemistry, and materia medica; the latter two they devote principally to the clinical teaching at the bedside. In both they have all the advantages they could possibly desire. The clinical teaching is all done at the bedside, and not in the amphitheatre. The surgeons visit their respective wards, the medical officers theirs, and so on throughout, until the whole is most systematically and well arranged. Each consumes several hours in the examination of patients, and every symptom is taken up separately and discussed, and the students rigidly quizzed on them. In nearly all instances the students are required to make the examinations, give the diagnosis, prognosis, and treatment, and any mistakes made are corrected. This system being very complete, entering into the pathology as well as the treatment, with all the bearings ably discussed, makes it peculiarly attractive and instructive. It is by this daily drilling, and from the ample opportunities offered, that one will generally find these students experts in diagnosis and treatment.

The hospitals I have been attending most usually are Guys, St. Thomas, St. Bartholomew's, and the London; these being more convenient to my place of abode. Guys is situated near the London bridge, and is a massive structure, with beautiful grounds attached. Among its physicians and surgeons are Sir William Gull, Habersham Pavy, Wilks, Moxon, Bryant (author of a valuable work on surgery), and Durham. Besides these, there are Hicks, Professor of Obstetrics, Baden, on the eye, and W. Loidlow Puroes, on the ear, with numerous assistants. Situated in a populous portion of the city, it is called one of the principal accident hospitals. The number of surgical operations performed here is very large. The eye department is considerable, and the aural clinic one of the largest in the world. It is conducted by Mr. Loidlow Puroes, from whom I obtained a clinical course of instruction through the kindness of Dr. Calhoun, of your city.

St. Thomas, situated on the banks of the Thames, opposite the Houses of Parliament, is a most magnificent building, and said to be among the most complete in the world. Its situation and pleasant surroundings make it a home attractive for patients. Among the medical men are Peacock, Murchison, Bristowe, Stone, Gerais, Croft, Sydney Jones, McCormock, and Liebreich. Mr. Murchison occupies the same position in England that Flint does in America. I have been attending his clinical lectures and teaching ever since I came, and find them very interesting. Among the surgeons, Mr. Croft especially stands very high with the students as a clinical teacher. Liebreich, on the eye, formerly of Paris, has quite an extensive clinic.

St. Bartholomew's is situated in the heart of the city, and is in one of the leading schools. Sir James Paget is the consulting surgeon, with Holden, Collenden, Savoury and Smith as surgeons. I have heard Sir James Paget several times on clinical lectures, and have "briefs" of them all. At the University are Erichisen, Berkley, Hill, Heath, Sir Wm. Jenner, Tillberry, Fox and others. At St. George's are Holmes, Barnes, on diseases of women, and others, whom I have not met. Neither have I had an opportunity of visiting King's College. And last, but not least, of these general hospitals, comes the London. Being situated in a laboring portion of the city, it is considered *the accident* hospital of London, besides all other departments being well filled. Like the others, the buildings are extensive and commodious, and the surroundings planned for every comfort of the patients. Americans especially seemed to show a decided preference for this hospital, judging from the number in attendance. Both at this, at St. Thomas, Guys, and possibly at some of the others, one can, by paying £10 ($50), be allowed a six months' practice in the hospital, including a drussership for three months in the medical portion and three in the surgical, besides extending the time as long as desired. This opportunity—with the privilege of examining hundreds of patients, both medical and surgical, in the wards, also with a daily average of twelve accidents brought in, which makes the surgery extensive—renders this hospital one of the best in London. It contains many men of prominence, too numerous to mention; among them Jonathan Hutchison, Andrew Clark, Hughlings Jackson, Sutton Toy, Morrell McKenzie, and others. But one of the best teachers is Mr. Smith, for a number of years house physician, and who has charge of several patients. His mode of clinical teaching and powers of pathology and diagnosis are not excelled by any whom I have met. It is with him that one of the finest opportunities is offered to study both the normal and diseased eye, as almost every patient is atrophized, and the eye examined with the ophthalmoscope.

Besides these general hospitals, every specialty is represented by several. Moorefield's Royal London Ophthalmic is said to be the largest ophthalmic hospital in the world It is estimated that they have 20,000 new cases every year. Go any day you may, except the Sabbath, and you will find several hundred patients in attendance. Operations until you almost tire of them are always on hand, besides all that could be desired in the number of cases for ophthalmic examinations and for testing refraction and accomodation. Bowmon, Soelberg, Wells, Critchett, Lawson, Coupon, Hutchison, etc., are to be found here. Near this is St. Marc's for fistula, piles and diseases of the rectum, with about eighty patients in daily attendance. One of the hopitals for diseases of women which I have visited is well attended, being about a daily average of one hundred. These coming but once a week makes the whole number in attendance very large. One of the hospitals for diseases of the skin to which I go has an average attendance of about one hundred and fifty each day.

Morrell McKenzie's throat hospital is the most attractive in that department in London. Private clinical instruction is given in courses of three months for a small sum, and those attending have the privilege of performing the various operations in the throat, such as the removal of tonsils, polipi, etc. The *chef d'clinique* for the past two years has been Dr. Samuel Johnson, a young man of Baltimore, Md., and who is especially skilled in that department. I was present a few days ago, when he removed from the larynx of a boy a foreign body, which had become encysted, and for which tracheotomy had some time previous been performed. It being impossible to remove it, an incision from the hyoid bone to the fifth trochial ring became absolutely necessary, in order to extricate it, embracing the three operations in that region. The body was successfully extricated, the larynx closed; recovery sure, with a good prospect of the normal voice, though the latter was hardly expected. Dr. Johnson sails in a few weeks, and will practice his specialty in Baltimore. I have also attended hospitals for stone, venereal troubles, and orthopœdic. At each of these one can see all that is desired in the way of clinics.

With Spencer Wells I will close my already too lengthy letter. I have seen him operate often. Every Wednesday, when the clock in the steeple strikes two, he is standing by his table, with sleeves rolled up and knife in hand. It would delight you to see the business-like manner and coolness with which he goes at his work. He doesn't seem to be in any hurry, but I have never seen him longer than twenty-five minutes from the time he began until his patient was in bed, though in none of the cases seen were there any complications. His success has been much greater than those operated on at the general hospitals, supposed to be due to surrounding influences. On one occasion I met Dr. Sims there, who is spending the summer in Europe. Mr. Wells' fatality is about one in every five cases. I do not know whether he selects his cases or not, but I rather think not. At the close of 1874 he had operated 337 times at his general hospital. The attendance at any of these special hospitals is free to medical men, except the throat. If a course is desired it must be paid for. If any degrees are desired, or one wishes to attend all the lectures of the college, both didactic and clinical, with also the privilege of the dissecting and microscopy, or special instruction, a fee of about $50 is required for six months, but to attend the hospital lectures and practice, including the operations, like other European scholars, the most of them charge nothing.

I could give you descriptions of the many operations I have seen, with the style and mode of operating, but I am afraid they would not interest you. Besides, I could furnish you with numerous "briefs" taken from the teachings of some of the most distinguished men here, but will no longer intrude on your time with medicine.

Have been so busily engaged that I have scarcely had an opportunity of seeing any places of historic interest. I visited for an hour the famous Tower of London, renowned for its tragic scenes of the past, yet still England's arsenal and the home of the crown jewels. Also snatched a few moments to see the Houses of Parliament. Gladstone and Disraeli still sway with their eloquence this august body. Also a short time near by in Westminister Abbey, where sleep the illustrious dead. In this solemn place rest the ashes of royalty, warriors, statesmen, poets, authors, physicians, divines, etc., in close proximity. On every side, in this city, is a busy, bustling throng. Along the spacious thoroughfares rush hourly uncounted thousands, in the busy pursuits of life. Here I have met the German, Russian, Canadian, Australian, and the black man, all in the pursuit of medicine.

In a few days I go to Paris to spend some time, and will try and write you something from your former home of study, of more interest than this. Excuse my length; only hoping you may be able to read it, I remain,

Yours, etc.,

W. S. K.

*London, August,* 1876.

